# Influence of Air Abrasion and Sonic Technique on Microtensile Bond Strength of One-Step Self-Etch Adhesive on Human Dentin

**DOI:** 10.1155/2015/368745

**Published:** 2015-03-23

**Authors:** Baraba Anja, Dukić Walter, Chieffi Nicoletta, Ferrari Marco, Sonja Pezelj Ribarić, Miletić Ivana

**Affiliations:** ^1^Department of Endodontics and Restorative Dentistry, School of Dental Medicine, University of Zagreb, Gundulićeva 5, 10 000 Zagreb, Croatia; ^2^Department of Paediatric Dentistry, School of Dental Medicine, University of Zagreb, Gundulićeva 5, 10 000 Zagreb, Croatia; ^3^Department of Dental Materials and Fixed Prosthodontics, University of Siena, Policlinico “Le Scotte”, Viale Bracci, 53100 Siena, Italy; ^4^Department of Oral Medicine and Periodontology, Clinical Hospital Centre, Faculty of Medicine, University of Rijeka, 51000 Rijeka, Croatia

## Abstract

The purpose of this *in vitro* study was to evaluate the microtensile bond strength of one-step self-etch adhesive to human dentin surface modified with air abrasion and sonic technique and to assess the morphological characteristics of the pretreated dentin surface. The occlusal enamel was removed to obtain a flat dentin surface for thirty-six human molar teeth. The teeth were randomly divided into three experimental groups (*n* = 12 per group), according to the pretreatment of the dentin: (1) control group, (2) air abrasion group, and (3) sonic preparation group. Microtensile bond strength test was performed on a universal testing machine. Two specimens from each experimental group were subjected to SEM examination. There was no statistically significant difference in bond strength between the three experimental groups (*P* > 0.05). Mean microtensile bond strength (MPa) values were 35.3 ± 12.8 for control group, 35.8 ± 13.5 for air abrasion group, and 37.7 ± 12.0 for sonic preparation group. The use of air abrasion and sonic preparation with one-step self-etch adhesive does not appear to enhance or impair microtensile bond strength in dentin.

## 1. Introduction

Achieving effective bonding to dentin is still a major challenge because of higher organic content of dentin, fluid pressure from the dentinal tubules, and the presence of the smear layer [1–3]. There are two main strategies used to create effective dentin bonding: etch-and-rinse adhesives which work by removing the smear layer with phosphoric acid, followed by the application of a primer and an adhesive and the self-etching adhesives which are composed of acidic primer, responsible for interaction with the smear layer, and an adhesive for infiltration of partially demineralized dental tissues. Acid etching of dentin, which removes the smear layer completely and demineralizes the subsurface [4], is an established and predictable clinical procedure, but features inherent to dentin conditioning can influence the bonding performance of adhesives [5]. Dentinal collagen exposed by an etch-and-rinse adhesive has been found to be highly vulnerable to hydrolytic and enzymatic degradation processes [6–8]. A promising approach to adhesion is the use of one-step self-etch adhesives that slightly demineralize the dentin surface and simultaneously provide resin infiltration [9]. When using self-etch adhesives, a hybrid layer is formed with the smear layer incorporated [4]. Self-etch adhesives can improve dentin bonding strength and provide adhesion to dentin comparable or even superior to bonds obtained with adhesive systems that advise acid-etching as a separate step of the bonding protocol [3, 4, 10]. Advantages of using self-etch adhesives include simplification of the bonding procedure, reduced technique sensitivity, since etching, priming, and bonding occur simultaneously [11], reduced risk of incomplete resin impregnation of the demineralized dentin, and reduced incidence of postoperative sensitivity [12]. Furthermore, self-etch adhesives are less sensitive to moisture control [13]. “Mild” self-etch adhesives (pH around 2) only partially dissolve the dentin surface, so that a substantial amount of hydroxyapatite remains available within a submicron hybrid layer [14], encapsulating and protecting the collagen [14, 15]. Adhesion is consequently obtained micromechanically through shallow hybridization and by additional chemical interaction of specific carboxyl/phosphate groups of functional monomers with residual hydroxyapatite [14]. Due to all their advantages, it is recommended for adhesive procedures to use a mild self-etch approach that appears to provide better long-term perspectives at dentin [16].

Different techniques are used for cavity preparation or modification of dentin surface which may result in distinct smear-layer features [17, 18]. The characteristics of a smear layer, obtained with different dentin pretreatments, influence strongly the effectiveness of self-etch adhesives and different bonding interactions could be expected [4, 19–21]. Dental adhesives were developed primarily for cavities prepared with burs. Due to newer different preparation techniques used in restorative dentistry, it is necessary to assess their effect on bonding of self-etch adhesives to dental hard tissues.

Air abrasion is a technique for cavity treatment which involves the use of aluminum oxide powder, in a fine stream of compressed air. As the particles collide with dentin, the kinetic energy of the particles is released, resulting in fracture of microscopic fragments [22]. In this way, air abrasion creates a roughened tooth surface which may make it more conducive to bonding. More recently, various types of sonic instruments were introduced for use in cavity preparation [23]. Sonic instruments might remove the smear layer from the dentin surface leaving it roughened.

The aim of this* in vitro *study was (1) to evaluate the microtensile bond strength of one-step self-etch adhesive to human dentin modified with air abrasion and sonic preparation and (2) to evaluate the morphological characteristics of the pretreated human dentin surface.

## 2. Materials and Methods

Thirty-six intact human molar teeth, with no restorations or caries lesions, extracted for periodontal or orthodontic reasons, were used in the experiment. After extraction, the teeth were thoroughly cleaned using brushes and curettes and stored in 1% chloramine solution at room temperature for one month until use. The teeth were randomly divided into three experimental groups (*n* = 12 per group), according to the dentin preparation: (1) control group; (2) air abrasion group; and (3) sonic preparation group.

### 2.1. Preparation of Specimens

The entire occlusal enamel was removed by sectioning with a circular diamond blade in an Isomet 1000 saw (Buehler, Düsseldorf, Germany), with a speed of 150–200 rpm under continuous water cooling to obtain flat dentin surface. In order to form smear layer on the bonding surface of dentin, the surface was hand polished with wet sandpapers of different grit size [24], from coarser to finer (400-, 600-, 1000-grit) for 60 seconds each. The bonding surface was washed with water and gently dried with an air syringe of a dental unit (Kavo Primus, 1058 S/TM/C/G, Biberach/Riss, Germany) prior to the pretreatment. One operator prepared all specimens with the particle abrasive instruments and sonic instruments. For the air-abrasive procedure, 50 *μ*m particles of aluminium oxide (Rondoflex, KaVo, Biberach, Germany) were used in a perpendicular direction to the dentin surface with 80 psi pressure for 15 seconds. In third group, the entire dentin surface was treated with a sonic instrument (KaVo Sonicflex 2003 L, KaVo, Biberach, Germany) with a diamond microtip number 32 for 15 seconds.

Ten teeth from each experimental group were selected for bonding procedure and subsequent microtensile bond strength testing. The remaining two teeth from each experimental group were used for scanning electron microscopy (SEM) analysis. Following the application of the adhesive system (G-bond, GC, Tokyo, Japan) according to the manufactures instructions (Table 1), a composite resin block (Gradia Direct, GC, Tokyo, Japan) 5 mm high was built up on the bonding surface, with the application of layers of the material not thicker than 2 mm, each one cured with a Bluephase LED light (Ivoclar Vivadent, Schaan, Liechtenstein, 1200 mW/cm^2^, soft start) for 20 seconds. The bonded specimens were stored in distilled water at 37°C for 24 hours. The bonded teeth were then embedded into acrylic resin (Orthocryl, Dentaurum, Ispringen, Germany). Afterwards, the embedded teeth were cross sectioned longitudinally with a diamond blade in Isomet 1000 saw (Buehler, Düsseldorf, Germany), with a speed of 150–200 rpm under continuous water cooling, to obtain multiple beam-shaped sticks, with a cross-sectional top of about 1 mm^2^. Beams were stored in at room temperature in sterile gauze soaked in saline. Before testing the bond strength, each beam was checked under the stereomicroscope (Olympus SZX-12, Optical Co, Europe, GMBH, Hamburg, Germany) to verify that the adhesive interface was perpendicular to its long axis. Only the beams with the adhesive interface perpendicular to the long axis were used in the experiment.

### 2.2. Testing Microtensile Bond Strength

The microtensile bond strength was tested with a universal testing machine (Triax Digital 50, Controls, Milano, Italy). Ends of each beam were glued with cyanoacrylate adhesive (Loctite gel, Henkel, Düsseldorf, Germany) to specially designed metal plates. Each beam was placed in the testing machine and the tensile load was applied at a crosshead speed of 0.5 mm/min, until the composite separated from the dentin. The load at the point of failure was recorded. Test beams were observed under a stereomicroscope to verify the failure mode (adhesive, cohesive, or both). Failures were classified as adhesive failure if the fracture site was entirely within the adhesive, mixed failure if the fracture site continued from the adhesive into either resin composite or dentin, and cohesive failure if the fracture occurred exclusively within the resin composite or dentin [25]. The cross-sectional area at the site of fracture was measured for each specimen to the nearest 0.01 mm with a digital caliper so the bond strength at failure (MPa) could be calculated.

### 2.3. SEM Evaluation

Two specimens from each experimental group were selected randomly after surface preparation and subjected to SEM examination, to observe the bonding surface. For the SEM analysis, specimens were cleaned in an ultrasonic bath for 5 minutes, gently decalcified with a 32% phosphoric acid (Bisco, Schaumburg, Illinois, USA) for 30 seconds, washed, and air dried. Samples were then dehydrated in an ascending ethyl alcohol series (25%, 50%, 70%, 80%, 90%, and absolute alcohol) with three baths for 5 seconds for each concentration, critical-point dried, and sputter coated with a gold layer in a vacuum apparatus (Polaron Range SC 7620, Quorum technology, Newhaven, UK). Specimens were observed under SEM (JSM-6060LV JEOL, Tokyo, Japan) operating at 16 kV and micrographs of dentin surfaces were taken at standardize magnifications.

### 2.4. Data Analysis

Data were statistically analyzed by a one way ANOVA, after confirming normal distribution of the results with Kolmogorov-Smirnov statistical test. Comparisons between groups were done using a Scheffe test at a 0.05 significance level. The statistical analysis was performed using Statistica 7.0 (StatSoft, Tulsa, OK, USA).

## 3. Results

### 3.1. SEM Observation of Dentin Surfaces

**Figure 1:**
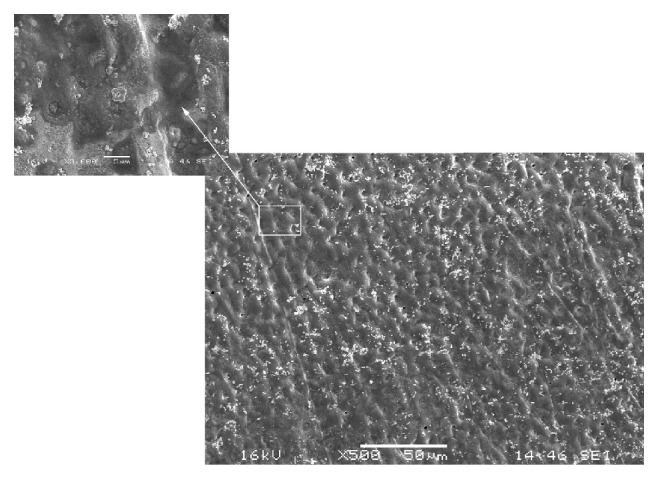
SEM (×500) showing dentin surface of the specimens in the control group. At higher magnification (×3000) intact smear layer can be observed.

The control group revealed a dentin surface with a small number of exposed dentin tubules and intact peritubular and intertubular dentin (Figure 1). It was also possible to verify an intact smear layer (Figure 1).

**Figure 2:**
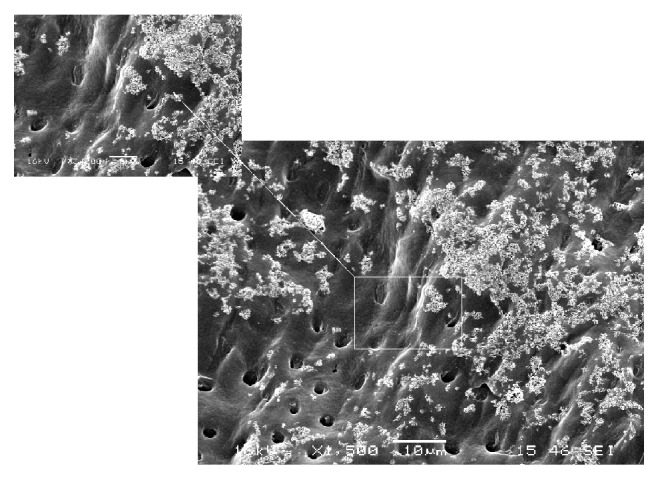
SEM (×1500, ×3000) showing dentin surface in air abrasion group.

**Figure 3:**
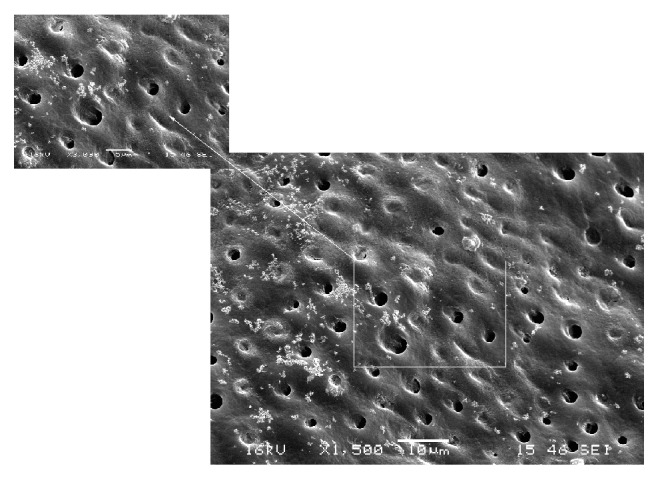
SEM (×1500, ×3000) showing dentin surface in sonic technique group.

Particle abrasion preparation procedure formed somewhat roughened dentin surface, with partially opened dentin tubules and intact peritubular and intertubular dentin (Figure 2). In the specimens prepared with the sonic technique, dentin surface was almost completely clean of smear layer with mostly open dentin tubules, but intact peritubular and intertubular dentin (Figure 3).

### 3.2. Microtensile Bond Strength

The number of specimens which were tested in the control, air abrasion, and sonic group was 64, 84, and 80, respectively. Means and standard deviations of microtensile bond strength expressed in MPa are shown in Table 2. There was no significant difference in microtensile bond strength between the three experimental groups (*P* > 0.05). In all groups, fractures were observed mostly between resin and dentin (adhesive failure) (Table 2).

## 4. Discussion

In this study, microtensile bond test was used to test the dentin adhesion of mild self-etch adhesive after three different methods of dentin preparation.* In vitro* studies examining the bond strength of restorative materials are important because they can predict their clinical behavior and long-term success. The advantages of such* in vitro* tests are their speed and simplicity, measuring just one experimental parameter and testing large number of specimens. Microtensile bond test, although possessing some limitations, remains useful as screening tools for new dental materials, adhesive approaches, and investigation of different experimental variables [26]. Reliable and accurate measurements of the microtensile bond test can be achieved if only the adhesive failures are considered for the bond strength calculation, which requires microscopic evaluation to verify the failure mode [27], and these requirements were fulfilled in the present study. Furthermore, reliability of bond strength data also depends on a number of adhesively failed specimens and a minimum of 30 specimens should be available for testing [27] and this study tested 43 specimens in the control group and 66 specimens in other two experimental groups. Although the teeth which were used for this study were collected and stored for one month until use, according to study of Santana et al. [28] this storage time does not influence the results of microtensile bond test. In order to create a standard and uniform smear layer, sandpapers of different grit sizes were used in the present study. This method provides a flat surface with fewer grooves and irregularities in comparison to rotary cutting instruments [29] and a uniform smear layer created can then be used for different surface treatments.

The results of this study showed that air abrasion and sonic technique did not influence the bond strength of one-step self-etch adhesive. SEM observations in previous studies showed that aluminium oxide air abrasion is able to produce roughened surface, increasing the surface area available for wetting and bonding by the adhesive resin [30, 31] which was confirmed with the micrographs in the present study. Similar appearance of dentin surface was observed after treatment using sonic technique. However, air abrasion and sonic technique did not increase microtensile bond strength in this study, which confirms the results of other studies [32, 33]. Considering that the surface roughness obtained with the air abrasion did not increase the adhesive bond strength in the present study, this characteristic is not the only factor influencing the bonding. Other factors also influence the adhesion: the chemical composition of the dentin surface and physical parameters [34]. Another factor which should be considered regarding mild self-etch adhesives is that they have micromechanical and chemical bond to hard dental tissues. Mild self-etching adhesives, such as the one used in the present study, do not completely expose collagen for micromechanical retention but provide an additional mechanism of ionic bonding [35]. 4-Methacryloxy-ethyl trimellitate anhydride (4-META), a demineralizing monomer with carboxylic groups, also found in the adhesive used in the present study, has been reported to improve adhesion to both enamel and dentin by establishing that ionic bond to calcium in hydroxyapatite [36]. Functional monomers in self-etching adhesives have also been shown to bond chemically to both dentin apatite and collagen [35]. The use of sonic instruments did not improve the bonding to dentin as well, although the surface was clean of smear layer. Considering that self-etch adhesives incorporate the smear layer in the hybrid layer [4] and that the formation of the resin tags in open dentinal tubules does not influence the bonding strength of self-etch adhesives [37], as the adhesive used in the present study, a possible conclusion is that these factors could explain why sonic technique did not improve the bonding to dentin.

According to the Soares et al. [38], aluminum oxide sandblasting procedure decreased the bond strength to bovine dentin which is not consistent with the results of the present study. Differences in the results can be explained by different samples employed in the studies. While Soares et al. [38] used bovine teeth for bond strength testing, in this study human teeth were used. Schilke et al. [39] reported that the density of dentin tubules is significantly greater in human dentin than in bovine dentin, which could explain different results. Furthermore, differences in the relative amounts of intratubular and intertubular dentine [40], or the nature of the intertubular matrix [41], in human and bovine teeth may result in differences in adhesive bond strength measurement. The use of air abrasion and sonic technique with one-step self-etch adhesive does not enhance or impair microtensile bond strength in dentin.

## 5. Conclusion

Beside conventional techniques using drills and burs, different techniques are used for preparation of hard dental tissues. According to the results of this study, the use of air abrasion and sonic technique with one-step self-etch adhesive does not appear to enhance or impair microtensile bond strength in dentin. Air abrasion and sonic technique can be used in combination with one-step self-etch adhesive as an alternative to conventional techniques.

## Figures and Tables

**Table 1:** Chemical composition and application procedure of G-bond, according to the manufacturer.

Chemical composition G-bond	Application mode G-bond
Acetone (40%), 4-META (15%), Water (20%), urethane dimethacrylate monomer (UDMA) (9%), triethylene glycol dimethacrylate (TEGDMA) (10%), phosphate monomer, 4-META: 4-methacryolxyethyl trimellitate anhydride; fumed silica filler, photoinitiators	Apply one coat of adhesive on dentin surface (dry or wet). Leave undisturbed for 10 s. Strong air-drying for 5 s. Light-cure for 10 s

**Table 2:** Microtensile bond strength values in MPa obtained for the different experimental groups and number of adhesive and cohesive failures.

Experimental group	Mean/MPa	SD	A^*^-failure	C^*^-failure
Control	35.3	12.8	43	21
Air abrasion	35.8	13.5	66	18
Sonic	37.7	12.0	66	14

A^*^: adhesive; C^*^: cohesive.
